# In Vivo Functional Investigation of LysC3 (Aspartokinase 3) Revealed Its Role in Priming the Biosynthesis of 1-Deoxynojirimycin in Mulberry

**DOI:** 10.3390/plants15152287

**Published:** 2026-07-26

**Authors:** Xinlei Wang, Guang Yang, Fengjie Gu, Shuaijun Zhang, Fangyuan Cao, Nan Chao, Li Liu

**Affiliations:** 1 Jiangsu Key Laboratory of Sericultural Biology and Biotechnology, School of Biotechnology, Jiangsu University of Science and Technology, Zhenjiang 212100, China; 241111802114@stu.just.edu.cn (X.W.); 231211802129@stu.just.edu.cn (G.Y.); 251111802132@stu.just.edu.cn (S.Z.); no.1lvtu@outlook.com (F.C.); 2 Xiajin County Forestry Development Center, Dezhou 253200, China; xjlyfzzx@163.com; 3 Key Laboratory of Silkworm and Mulberry Genetic Improvement, Ministry of Agriculture and Rural Affairs, Sericultural Research Center, Chinese Academy of Agricultural Sciences, Zhenjiang 212100, China

**Keywords:** 1-deoxynojirimycin, biosynthesis, aspartokinase, MmLysC3, Mulberry

## Abstract

Aspartokinase (LysC) has been proposed as a key enzyme involved in 1-deoxynojirimycin (1-DNJ) biosynthesis in mulberry, yet direct functional evidence remains limited. In this study, we cloned and functionally characterized *MmLysC3*, a candidate aspartokinase gene from mulberry (*Morus multicaulis*), and investigated its role in 1-DNJ biosynthesis. Domain architecture analysis confirmed the presence of both the conserved AA_kinase catalytic domain and two ACT regulatory domains, placing MmLysC3 within the class I aspartokinase family. Expression profiling revealed that *MmLysC3* exhibits tissue-preferential expression, with higher transcript levels in buds and young leaves, spatially coinciding with active 1-DNJ accumulation sites. Correlation analysis across ten mulberry varieties demonstrated a significant positive association between LysC3 expression and 1-DNJ content (Spearman’s r = 0.6743, *p* = 9.04 × 10^−5^). Critically, transient overexpression of *MmLysC3* in mulberry leaves resulted in a marked increase in 1-DNJ content, with the highest-accumulating line reaching approximately twice that of the control. These findings provide the first functional evidence that LysC3 acts as a positive rate-limiting enzyme in the aspartate-derived branch of the 1-DNJ biosynthetic pathway in mulberry, bridging the gap between transcriptomic predictions and functional validation.

## 1. Introduction

Mulberry (*Morus* spp.) is a perennial woody plant of significant economic and medicinal value, with its leaves serving as the exclusive feed for silkworms and its fruits being consumed as health-promoting foods [1]. Different parts of mulberry (*Morus alba* L.) have been extensively used in traditional Chinese medicine, owing to their rich repertoire of secondary metabolites, including flavonoids, stilbenoids, and alkaloids [2,3].

Among these, 1-deoxynojirimycin (1-DNJ)—a polyhydroxylated piperidine alkaloid—has garnered substantial attention due to its potent inhibitory activity against intestinal α-glucosidases, thereby suppressing postprandial blood glucose spikes [4,5]. Beyond glycemic control, 1-DNJ exhibits multiple beneficial bioactivities, including enhancing insulin sensitivity, promoting cholesterol efflux, attenuating high glucose-induced cellular senescence, reducing hepatic lipid accumulation, and even inhibiting HIV glycoprotein processing [6,7,8,9]. With the global prevalence of diabetes projected to reach 700 million by 2045 [10], plant-derived natural α-glucosidase inhibitors like 1-DNJ offer promising therapeutic alternatives with fewer side effects than synthetic drugs.

Despite its pharmacological importance, the biosynthetic pathway of 1-DNJ in mulberry remains only partially elucidated. Two distinct precursor routes have been proposed: one originating from L-lysine (derived from aspartate) and another from glucose via 2-amino-2-deoxy-D-mannitol (ADM) [11,12,13,14]. Recent transcriptomic analyses in Morus alba have outlined a putative aspartate-derived pathway, identifying aspartokinase (LysC) as the initial committing enzyme that catalyzes the phosphorylation of aspartate to aspartyl-4-phosphate [13]. However, direct experimental evidence linking LysC activity to 1-DNJ accumulation has been lacking [15].

Aspartokinase is a key regulatory enzyme in the biosynthesis of essential amino acids (lysine, threonine, methionine, and isoleucine) across bacteria, fungi, and plants, and is subject to sophisticated feedback inhibition by downstream metabolites [16,17,18,19]. Whether this enzyme plays a similarly regulated role in 1-DNJ biosynthesis, and how its expression correlates with alkaloid accumulation in plants, remains unknown. Furthermore, recent breakthroughs have identified additional components of the 1-DNJ biosynthetic network, including the ADM dehydrogenase MnGutB1 (catalyzing the oxidation of ADM to nojirimycin) [14], the cytochrome P450 monooxygenase MaCYP82C169 (mediating C-7 hydroxylation) [20], and an HDG11-mediated transcriptional regulatory network integrating cytokinin and ABA signals [21]. Yet, the functional contribution of the aspartokinase branch within this multi-pathway system is still underexplored.

In this study, we cloned *MmLysC3* from a cultivated mulberry variety, comprehensively analyzed its expression profiles across diverse germplasm and tissues, and performed transient overexpression to directly test its regulatory impact on 1-DNJ accumulation. Our results provide the first functional evidence that LysC3 acts as a positive rate-limiting factor in 1-DNJ biosynthesis, offering new insights into the metabolic regulation of this valuable alkaloid and laying the groundwork for pathway engineering.

## 2. Results

### 2.1. Cloning and Characterization of LysC in Mulberry

**Figure 1:**
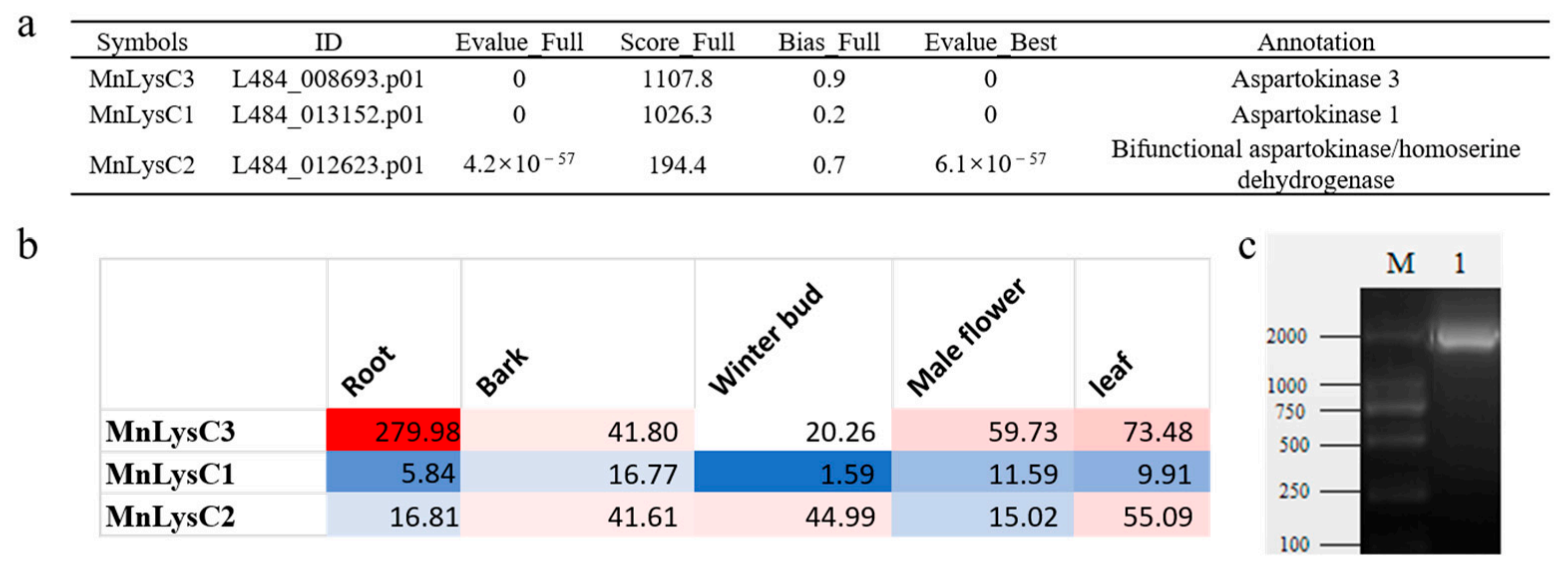
Genome screening and cloning of LysC genes in mulberry. (**a**) HMMER search results using the *Morus* genome database (MorusDB), showing the identification of three candidate LysC genes (*MnLysC1*, *MnLysC2*, *MnLysC3*) with their protein ID, full-length score, bias value and E-value obtained from genome-wide homologous identification. (**b**) Expression profiles of the three candidate LysC genes in different organs (root, bark, winter bud, male flower, and Leaf) based on transcriptome data (FPKM values) red shading represents high gene expression abundance, and blue shading represents low expression abundance. (**c**) Agarose gel electrophoresis of the cloned *MmLysC3* coding sequence. M, DNA marker; lane 1, MmLysC3 amplicon.

Genome-wide screening of LysCs in mulberry using HMMERsearch indicated three candidate LysC homologs in mulberry (Figure 1a). These three LysCs were named LysC1-3 according to the annotation information. In addition, based on the transcriptome data of different organs of mulberry, *LysC3* showed higher expression levels in examined organs and showed an obvious preference for roots (Figure 1b). Then we cloned the full CDS of *MmLysC3* in *Morus multicaulis* (Figure 1c). The *MmLysC3* CDS has a length of 1683 bp and encodes 560 amino acids. The putative MmLysC3 has predicted values of PI = 8.54 and Mw = 66.57 kD. This sequence has been submitted to GenBank (Accession Number MT427780).

### 2.2. Alignment and Physiology Analysis of LysC in Plants

**Figure 2:**
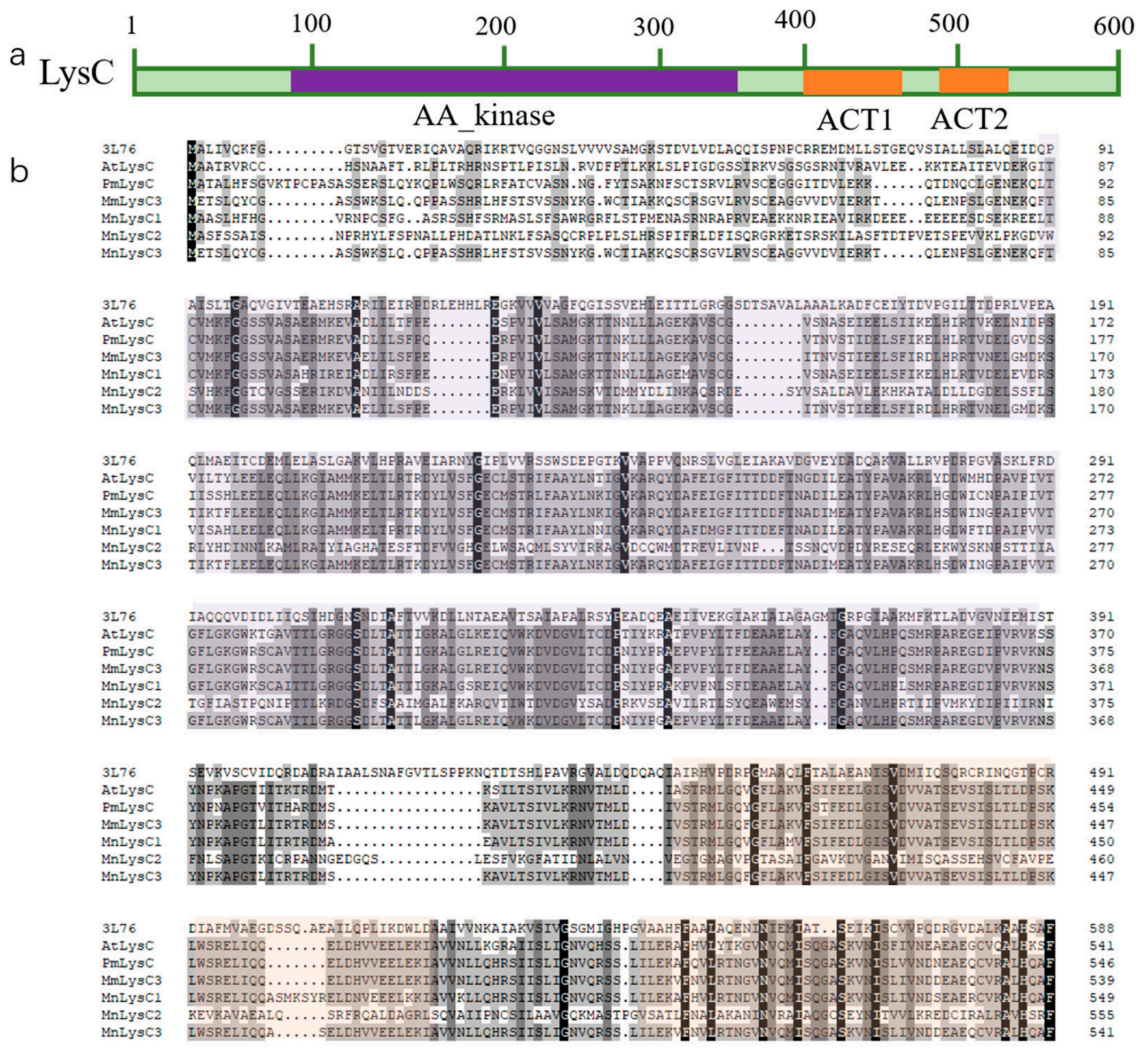
Amino acid sequence analysis of MmLysC3 and other plant LysC proteins. (**a**) Schematic diagram of the MmLysC3 protein based on motif search results, showing the domain architecture: AA_kinase domain (approximately positions 1–300), ACT1 domain (~300–400), ACT2 domain (~400–500), and C-terminal LysC domain (~500–600). (**b**) Multiple sequence alignment of MmLysC3 with other LysC proteins from Synechocystis sp. PCC6803 (3L76), Arabidopsis thaliana AtLysC (CAA67376.1), and Prunus mume PmLysC (XP_008231032.1). The AA_kinase domain is highlighted with a reddish shadow, and the ACT1 and ACT2 domains are highlighted with a yellow shadow, indicating conserved regions across species.

**Figure 3:**
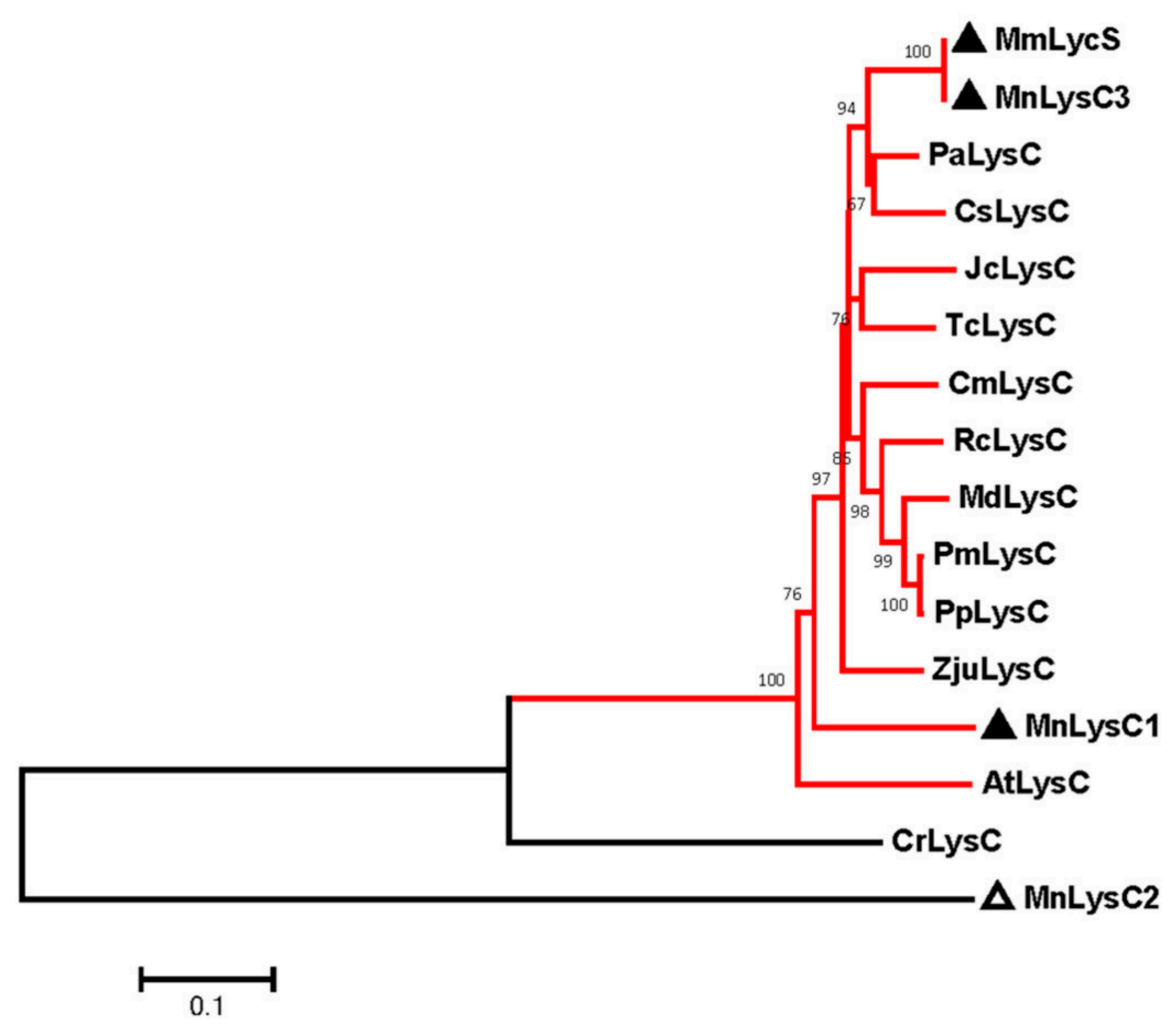
Phylogenetic analysis of LysCs. *Morus multicaulis* MmLysC3 (MT427780), *Morus notabilis* MnLysC1 (L484_013152), MnLysC2 (L484_012623), MnLysC3 (XP_024021164.1), *Parasponia andersonii* PaLysC (PON68853.1), *Prunus mume* PmLysC (XP_008231032.1), *Prunus persica* PpLysC (XP_020415398.1), *Cannabis sativa* CsLysC (XP_030484355.1), *Ziziphus jujuba* ZjuLysC (XP_015893856.1), *Malus domestica* MdLysC (XP_008362086.1), *Castanea mollissima* CmLysC (KAF3961984.1), *Jatropha curcas* JcLysC (XP_012067342.1), *Rosa chinensis* RcLysC (XP_024182251.1), *Theobroma cacao* TcLysC (XP_007032014.2), *Chlamydomonas reinhardtii* CrLysC (XP_001698576.1), *Arabidopsis thaliana* AtLysC (CAA67376.1).

All the characterized LysCs have an N-terminal amino acid kinase domain (Pfam 00696) connected to a regulatory domain containing a variable number of two ACT domains [Pfam 01842; small regulatory domains initially found in AK(Aspartokinase), chorismite mutase and TyrA (prephenate dehydrogenase)] as indicated by the schematic diagram. Alignment of MmLysC3, MnLysCs and AtLysC, PmLysC and LysC from *Synechocystis* sp. PCC 6803 showed to be relatively conserved in the AA-kinase domain and two ACT domains, belonging to class I which contains the homodimeric AKs with two ACT domains per monomer (Figure 2) [22]. Phylogenetic analysis showed that *MmLysC3* and MnLysC1 cluster with other LysCs while *MnLysC2* is distinct with these LysCs (Figure 3). These results indicated *MmLysC3*, *MmLysC3* and *MnLysC1s* should be the bona fide LysCs in mulberry. Given the low expression level of *MnLysC1*, *LysC3* is likely to play a dominant role in mulberry.

### 2.3. Co-Expression of LysC3 and 1-DNJ in Different Plant Organs

**Figure 4:**
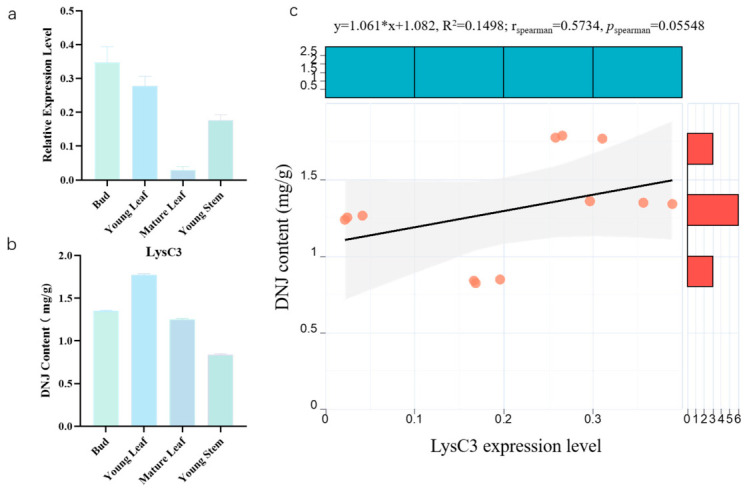
Tissue-specific expression of *LysC3* and its correlation with 1-DNJ content in mulberry. (**a**) Relative expression level of *LysC3* in bud, young leaf, mature leaf, and young stem tissues. (**b**) 1-DNJ content (mg/g fresh weight) in the corresponding tissues. (**c**) Scatter plot showing the correlation between *LysC3* relative expression level and 1-DNJ content, orange dots represent individual tissue samples, the light gray shaded area indicates the 95% confidence interval of linear regression. The linear regression equation is y = 1.061x + 1.082, Spearman’s rank correlation coefficient r = 0.5734, and *p* = 0.05548, indicating a significant positive correlation.

Expression levels of *MmLysC3* in different organs (tissues) were detected. *MmLysC3* showed higher expression levels in buds and tender leaves, which corresponded with the 1-DNJ distribution in these plant organs (Figure 4a,b). Correlation analysis revealed a positive correlation between *MmLysC3* expression and 1-DNJ content across different organs, although it did not reach statistical significance (R^2^ = 0.1498, r_spearman_ = 0.5734, *p*_spearman_ = 0.05548), suggesting a trend that LysC3 may be involved in the biosynthetic pathway of 1-DNJ in mulberry. The marginal significance (*p*_spearman_ = 0.05548) is likely attributable to the limited sample size (*n* = 4 organ types) for correlation analysis, as the statistical power for detecting a moderate-to-strong correlation (r_spearman_ = 0.5734) with only four data points is inherently constrained. To further validate this trend and assess whether the positive correlation holds across a broader genetic background, we extended the analysis to a panel of ten mulberry varieties.

### 2.4. Co-Expression of LysC3 and 1-DNJ in Different Mulberry Varieties

**Figure 5:**
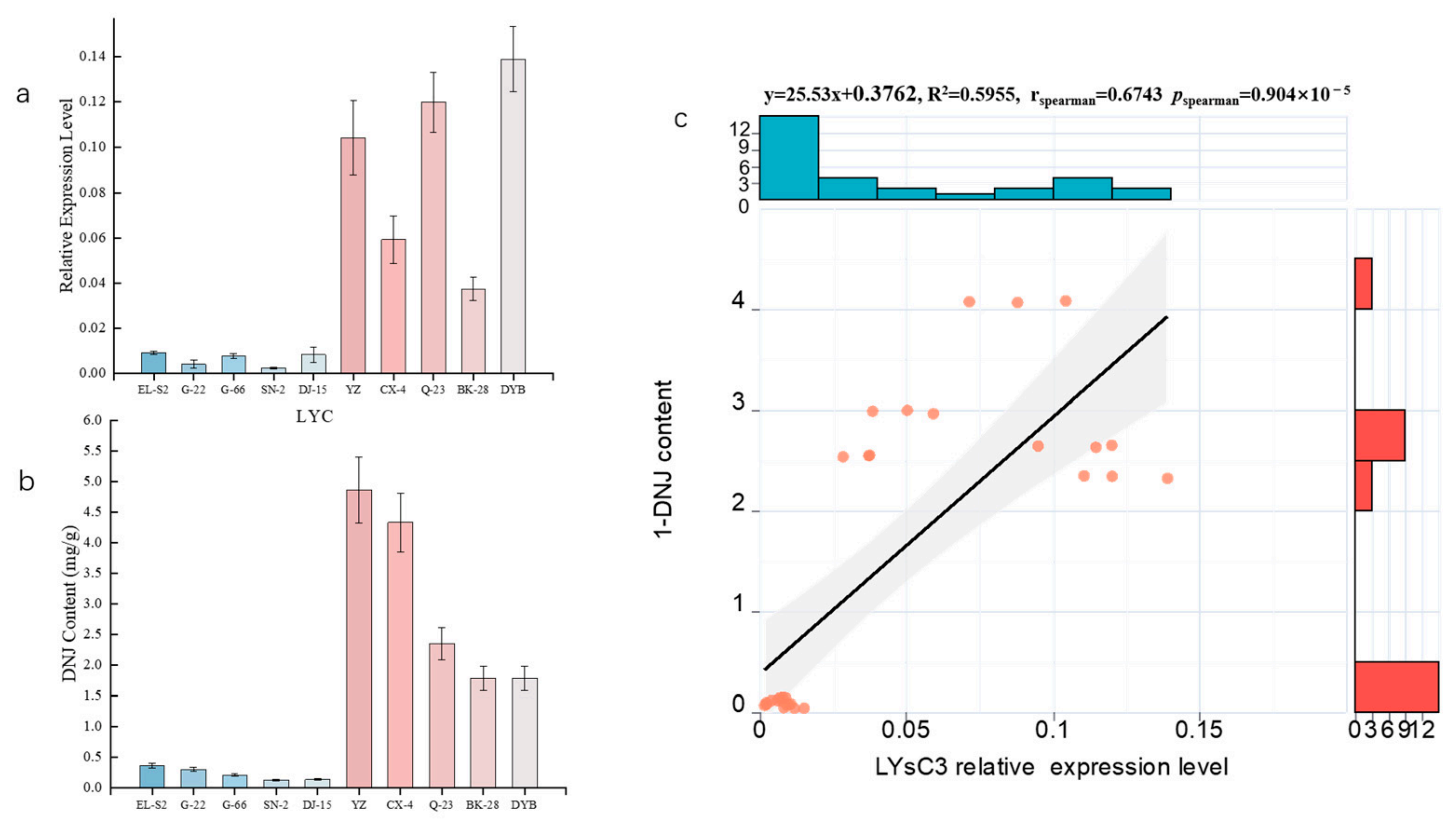
Co-expression analysis of *LysC3* and 1-DNJ content across ten different mulberry varieties. (**a**) Relative expression levels of *LysC3* in ten mulberry cultivars (EL-S2, G-22, G-66, SN-2, DJ-15, YZ, CX-4, Q-23, BK-28, and DYB). (**b**) 1-DNJ content (mg/g) in the corresponding varieties. (**c**) Correlation analysis of *LysC3* expression and 1-DNJ accumulation based on the data from the ten varieties. orange dots represent individual tissue samples, the light gray shaded area indicates the 95% confidence interval of linear regression. The linear regression equation is y = 25.53x + 0.3762, with R^2^ = 0.5955, Spearman’s rank correlation coefficient r = 0.6743, and *p* = 9.04 × 10^−5^, indicating a significant positive correlation. The consistent positive association between transcript abundance and metabolite level across diverse genetic backgrounds further supports the role of *LysC3* in 1-DNJ biosynthesis.

We next examined the correlation between *LysC3* expression and 1-DNJ content across ten mulberry cultivars with diverse genetic backgrounds. Substantial variation in both parameters was observed among the cultivars. Varieties with higher *LysC3* transcript levels (e.g., DYB, BK-28) generally accumulated greater amounts of 1-DNJ, whereas low-expressing varieties (e.g., EL-S2, G-22) exhibited correspondingly lower 1-DNJ levels (Figure 5a,b). Correlation analysis across these ten varieties revealed a significant positive association between *LysC3* expression and 1-DNJ accumulation (R^2^ = 0.5955, r_spearman_ = 0.6743, *p*_spearman_ = 9.04 × 10^−5^) (Figure 5c). This variety-level correlation reinforces the functional link between *LysC3* expression and 1-DNJ production across a broad genetic background, further supporting the role of LysC3 in 1-DNJ biosynthesis.

### 2.5. Overexpression of LysC3 Promote 1-DNJ Accumulation in Mulberry Leaves

To functionally validate the role of LysC3 in 1-DNJ biosynthesis, we performed transient overexpression of *MmLysC3* in mulberry leaves via Agrobacterium-mediated infiltration. Five independent transgenic lines (OE-LysC3) with significantly elevated transcript levels compared to the empty-vector control (CK) were obtained. Quantitative analysis revealed that 1-DNJ content in these overexpression lines was markedly increased, with the highest-accumulating line reaching approximately twice the level observed in the CK. These results provide direct functional evidence that *LysC3* encodes a bona fide aspartokinase that catalyzes a key rate-limiting step in the 1-DNJ biosynthetic pathway in mulberry.

## 3. Discussion

In this study, we cloned and functionally characterized MmLysC3, a candidate aspartokinase from mulberry, and systematically investigated its role in 1-DNJ biosynthesis. Phylogenetic analysis placed MmLysC3 within a clade containing bona fide LysCs from Morus notabilis, Ulmaceae, and Malvales, while domain architecture analysis confirmed the presence of both the conserved AA_kinase catalytic domain and the two ACT regulatory domains characteristic of class I aspartokinases [22]. These structural features are consistent with the enzyme’s proposed role as a feedback-regulated aspartokinase in primary metabolism, and our data further extend its function to secondary metabolite biosynthesis.

**Figure 6:**
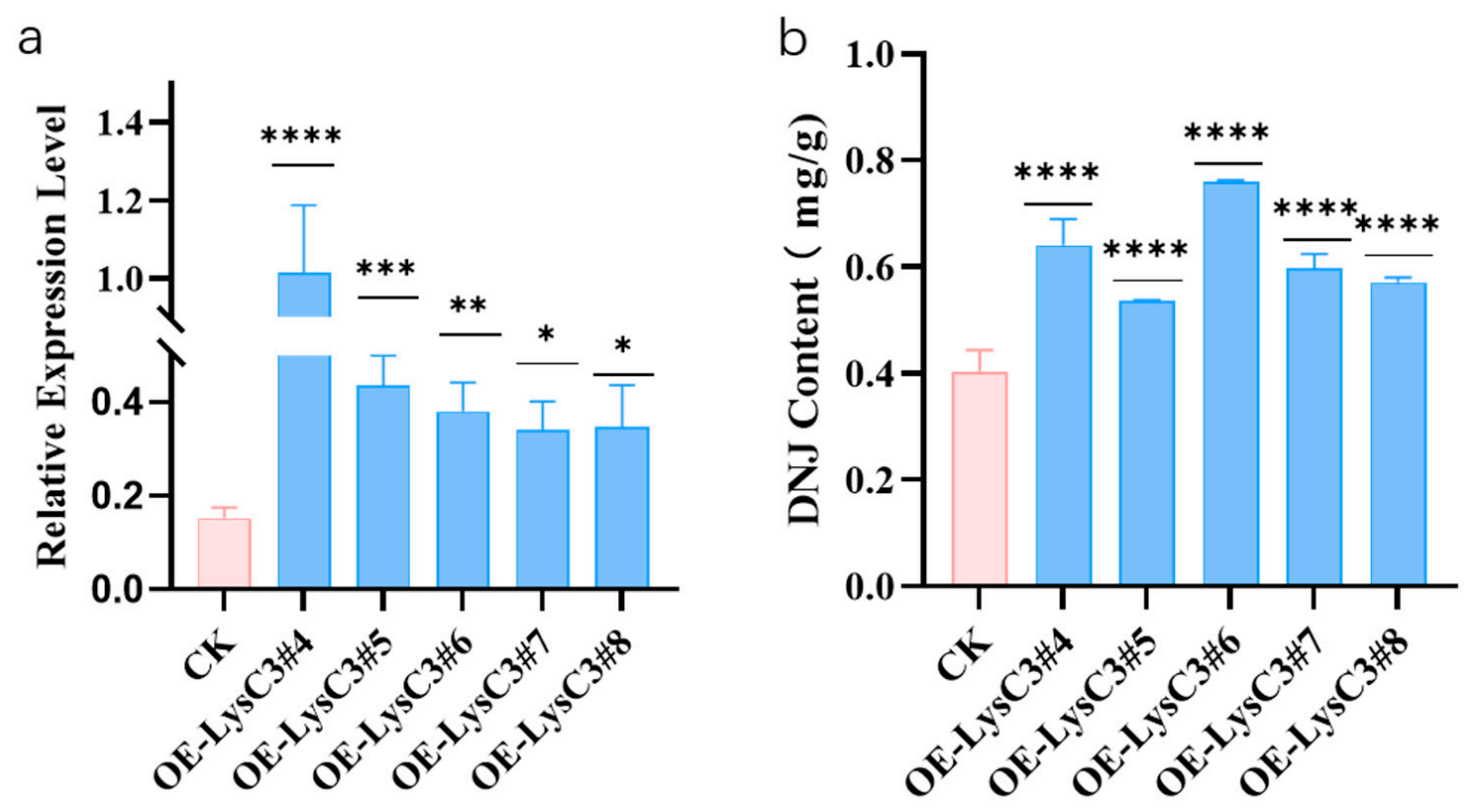
Functional validation of *LysC3* through overexpression in mulberry. (**a**) Relative expression level of LysC3 determined by qRT-PCR in the untransformed control (CK) and five independent LysC3-overexpression lines (OE-LysC3 #4, #5, #6, #7, and #8). (**b**) 1-DNJ content (mg/g fresh weight) in CK and five OE-LysC3 transgenic lines. Asterisks denote statistically significant differences relative to the CK group: (* *p* < 0.05, ** *p* < 0.01, *** *p* < 0.001, **** *p* < 0.0001).

The involvement of LysC3 in 1-DNJ biosynthesis is supported by three converging lines of evidence. First, *MmLysC3* exhibited a tissue-preferential expression pattern that positively correlated with 1-DNJ accumulation, with both transcript and metabolite peaking in young leaves and buds (Figure 4). This spatial coincidence is biologically significant, as young leaves and buds are the primary sites of active secondary metabolism in mulberry. Although the organ-level correlation was marginally significant *(p_spearman_* = 0.05548) due to the limited sample size (n = 4), the consistent positive trend supports a functional role for LysC3. Second, the positive correlation was recapitulated across ten diverse mulberry varieties (Figure 5), with high-expressing varieties (DYB, BK-28) showing elevated 1-DNJ content and low-expressing varieties (EL-S2, G-22) exhibiting reduced DNJ accumulation. The significant correlation at the variety level (r_spearman_ = 0.6743, *p_spearman_* = 9.04 × 10^−5^) confirms that natural genetic variations affecting *LysC3* expression are associated with corresponding differences in 1-DNJ content. Third, and most critical, the transient overexpression of *MmLysC3* consistently elevated 1-DNJ levels to approximately twice those of the control (Figure 6), providing direct causal evidence that LysC3 activity drives DNJ accumulation. Collectively, these findings establish that LysC3 acts as a positive rate-limiting enzyme in the aspartate-derived branch of 1-DNJ biosynthesis.

While the overall correlation was significantly positive, the relationship was not perfectly linear (R^2^ = 0.5955), suggesting that additional regulatory layers modulate 1-DNJ accumulation beyond LysC3 transcription. One likely mechanism is the well-documented feedback inhibition of aspartokinase activity by downstream amino acids (lysine, threonine) or by 1-DNJ itself, which may fine-tune enzyme activity post-translationally [18]. Furthermore, recent discoveries of the glucose-derived ADM pathway—involving MnGutB1 dehydrogenase [14] and MaCYP82C169 hydroxylase [20]—indicate that 1-DNJ biosynthesis is a metabolic network with multiple entry points [13]. The recent discovery of an HDG11-mediated transcriptional cascade that integrates cytokinin and ABA signals to regulate the glucose-derived DNJ pathway suggests that DNJ biosynthesis is subject to complex multilayered regulation [21]. The aspartokinase branch and the glucose-derived branch are not mutually exclusive but may converge at downstream steps, with relative flux through each pathway depending on precursor availability, developmental stage, and environmental cues.

In summary, we have demonstrated that MmLysC3 primes the aspartate-derived branch of 1-DNJ biosynthesis in mulberry. Our findings bridge the gap between transcriptomic predictions [16] and functional validation, opening new avenues for exploring the cross-talk between primary amino acid metabolism and secondary alkaloid production, and laying the groundwork for metabolic engineering of this pharmacologically valuable alkaloid.

## 4. Materials and Methods

### 4.1. Plant Materials

Samples of different organs (tissues), including buds, leaves and stem were collected from a ten-year-old Zhong Sang 9703 mulberry tree grown in the National Germplasm Mulberry Plantation of Zhenjiang, Institute of Sericulture, Chinese Academy of Agricultural Sciences. Additionally, tender leaves from ten representative varieties (EL-S2, G-22, G-66, SN-2, DJ-15, YZ, CX-4, Q-23, BK-28, and DYB) were selected for detailed correlation analysis between *LysC3* expression and 1-DNJ content. All collected samples were immediately frozen in liquid nitrogen and stored at −80 °C until use.

### 4.2. RNA Extraction and cDNA Synthesis

RNA was extracted from mulberry samples according to the manual provided by the RNA extraction kit RN34 from Aidelab (Beijing, China) and the quality was detected by both electrophoresis and Nanodrop One (Thermo Scientific, Wilmington, NC, USA). cDNA was synthesized after Dnase digestion (Promega, Madison, WI, USA), with the Reverse Transcriptase M-MLV (RNase H+) (Takara, Dalian, China), according to the manufacturer’s protocol using a p(dT)18 primer.

### 4.3. Genome-Wide Identification and Cloning of LysC3 in Mulberry

LysC sequences from different plants were collected using accession numbers according to reported studies and NCBI blast results (Figure 1a). These sequences were submitted to a HMMER search as query sequences with default parameters using MorusDB online (https://morus.swu.edu.cn/morusdb) [23,24]. Top hits were screened as candidate LysCs in Morus. The expression levels of candidate LysCs were further evaluated based on the assembled transcriptome data in MorusDB. Based on CDS (coding sequence) sequences information of *LysC3* in *Morus notabilis* C.K. Schneid, primers LysC3-F2: 5′-TGGAGACCTCATTGCAGTATTG-3′ and LysC3-R2: 5′-TTATAACAATG AGAGAACAGCACCG-3′ were used for cloning LysC3 in *Morus multicaulis*. The primers were synthesized by Sangon Biotech (Shanghai, China). The standard three-step PCR process with an annealing temperature at 58 °C was adopted to amplify *MmLysC3*. The PCR products were purified using SanPrep Column DNA Gel Extraction Kit (Sangon Biotech, Shanghai, China) and then were cloned into the PMD18-T vector (Takara, Dalian, China), propagated in *Escherichia coli* DH5α. The inserts were confirmed by sequencing. Sequences were deposited at GenBank.

### 4.4. Alignment and Phylogenetic Analysis of LysC in Plants

Alignment of LysCs in mulberry and other LysCs in Arabidopsis, Prunus mume and *Synechocystis* sp. PCC 6803 was performed using DNAman 8.0 (Lynnon BioSoft, Vaudreuil-Dorion, QC, Canada) with default parameters. Motifs and pfam domains were also scanned using the Motif Search online tool (https://www.genome.jp/tools/motif/). In addition, the phylogenetic tree was constructed using Mega 7.0 with the maximum-likelihood method [25,26]. The phylogenetic tree was assessed by boot-strapping using 1000 bootstrap replicates and marked above nodes only if greater than 50. The JTT substitution model and G+I rates among sites model were selected as parameters for building the tree.

### 4.5. MmLysC3 Gene Expression Profiles

The primers used for qRT-PCR (quantitative real-time PCR) were designed based on the cloned CDS sequence of *MmLysC3*. The actin gene β-actin was used as a reference gene. Primers are as follows: LysC3-QF: 5′-GCGACATCTCTGAACTGGGT-3′; LysC3-QR: 5′-GACAACTTCCCGTCGGACAA3′; β-actin-F: 5′-CATTGTCTT GTCTGGTT-3′; β-actin-R: 5′-TCATCATAC TCCGACTTTGC-3′. The reaction system consisted of 20 μL containing 10 μL 2× SYBR Master mix (Vazyme, Nanjing, China), 1.0 μL upstream and downstream primers respectively, 2 μL cDNA template (40 ng/μL), and 6 μL ddH2O. Three biological replicates with three technical replicates respectively were performed using ABI StepOnePlus™ Real-Time PCR System (Applied Biosystems, Foster City, CA, USA). The relative amount of each gene is calculated according to the formula (2^△△Ct)^.

### 4.6. Transient Overexpression of MmLysC3 in Mulberry Leaves

The full-length CDS of *MmLysC3* was cloned into the plant overexpression vector pCAMBIA2300 under the control of the CaMV 35S promoter. The recombinant plasmid was introduced into *Agrobacterium tumefaciens* strain GV3101 by electroporation. For transient transformation, Agrobacterium cultures were grown in LB medium containing appropriate antibiotics at 28 °C with shaking to an OD_600_ of approximately 0.6–0.8. Cells were harvested by centrifugation, washed, and resuspended in infiltration buffer (10 mM MgCl_2_, 10 mM MES, pH 5.6, and 200 μM acetosyringone). The bacterial suspension was infiltrated into fully expanded young leaves of mulberry using a needleless syringe. The empty vector was infiltrated as a negative control (CK). Infiltrated plants were maintained under normal growth conditions for 48–72 h. Leaf tissues from infiltrated areas were harvested for RNA extraction and 1-DNJ content determination. Five independent transgenic lines with confirmed overexpression of *MmLysC3* were selected for further analysis (Figure 6).

### 4.7. Determination of 1-DNJ Content in Mulberry

The 1-DNJ content in mulberry samples was determined using a pre-column derivatization reversed-phase high-performance liquid chromatography with fluorescence detection (HPLC-FLD) method [11,20]. Dried mulberry powder (0.1 g) was extracted twice with 10 mL of 0.05 M HCl, and the pooled supernatants were derivatized with 9-fluorenylmethyl chloroformate (FMOC-Cl) in 0.4 mol/L borate-potassium buffer (pH 8.5) consisting of boric acid and potassium chloride at room temperature for 20 min. After terminating the reaction with glycine, the derivatized product (DNJ-FMOC) was separated on a reversed-phase C18 column (250 × 4.6 mm, 5 μm) at 30 °C, using a mobile phase of acetonitrile and 0.1% aqueous acetic acid (55:45, *v*/*v*) at a flow rate of 1.0 mL/min. Fluorescence detection was performed at excitation and emission wavelengths of 254 nm and 322 nm, respectively. Quantification was achieved using an external standard curve (linear range: 0.3–30 μg/mL), and 1-DNJ content was expressed as μg/g dry weight.

### 4.8. Statistical Analysis

All experiments were performed with at least three biological replicates. Data are presented as mean ± standard deviation (SD). Statistical comparisons between overexpression lines and the control were performed using Student’s *t*-test (two-tailed). Correlation analyses between *LysC3* expression levels and 1-DNJ content were conducted using Spearman’s rank correlation coefficient. A *p*-value < 0.05 was considered statistically significant. All statistical analyses were performed using GraphPad Prism (version 8.0) or SPSS (version 22.0) software.

## 5. Conclusions

In this study, we cloned and functionally characterized *MmLysC3* from mulberry and demonstrated its positive role in 1-DNJ biosynthesis through expression profiling and transient overexpression experiments. *MmLysC3* expression showed a significant positive correlation with 1-DNJ content across mulberry varieties, and its overexpression led to an approximately two-fold increase in 1-DNJ accumulation, confirming that LysC3 catalyzes a key rate-limiting step in the aspartate-derived branch of the 1-DNJ biosynthetic pathway. These findings establish LysC3 as a positive regulator of 1-DNJ biosynthesis, provide valuable genetic resources for marker-assisted breeding, and demonstrate the feasibility of metabolic engineering for enhancing the production of this pharmacologically valuable alkaloid in mulberry.

## Data Availability

All data presented in this study are fully available within the article.
